# Regulation of immune cells by miR-451 and its potential as a biomarker in immune-related disorders: a mini review

**DOI:** 10.3389/fimmu.2024.1421473

**Published:** 2024-07-15

**Authors:** Fei-xiang Wang, Zu-an Shi, Guo Mu

**Affiliations:** ^1^ Department of Anesthesiology, The Affiliated Hospital, Southwest Medical University, Luzhou, Sichuan, China; ^2^ Anesthesiology and Critical Care Medicine Key Laboratory of Luzhou, Southwest Medical University, Luzhou, Sichuan, China; ^3^ Department of Anesthesiology, Zigong Fourth People’s Hospital, Zigong, Sichuan, China

**Keywords:** MiR-451, microRNAs, immune cells, biomarker, immune-related disorders

## Abstract

In 2005, Altuvia and colleagues were the first to identify the gene that encodes miR-451 in the human pituitary gland, located in chromosome region 17q11.2. Subsequent studies have confirmed that miR-451 regulates various immune cells, including T cells, B cells, microglia, macrophages, and neutrophils, thereby influencing disease progression. The range of immune-related diseases affected encompasses various cancers, lymphoblastic leukemia, and injuries to the lungs and spinal cord, among others. Moreover, miR-451 is produced by immune cells and can regulate both their own functions and those of other immune cells, thus creating a regulatory feedback loop. This article aims to comprehensively review the interactions between miR-451 and immune cells, clarify the regulatory roles of miR-451 within the immune system, and assess its potential as both a therapeutic target and a biomarker for immune-related diseases.

## 1 Introduction

Immune cells are pivotal in safeguarding human health and warding off disease incursions. Serving as essential elements of the immune system, they identify and eradicate pathogens that breach the human body, including bacteria, viruses, and cancerous cells. The spectrum of immune cells encompasses a variety of types, notably lymphocytes (T cells and B cells), macrophages, dendritic cells, and natural killer cells, among others (1–3). Through their distinct mechanisms and pathways, these cells collaborate to preserve the body’s internal equilibrium and shield it from external threats. For instance, Immune cells play critical roles in infection defense, tumor immunosurveillance, and the modulation of inflammatory responses (4–6). The effective performance of these functions is vital for maintaining health and forestalling diseases.

**Figure 1:**
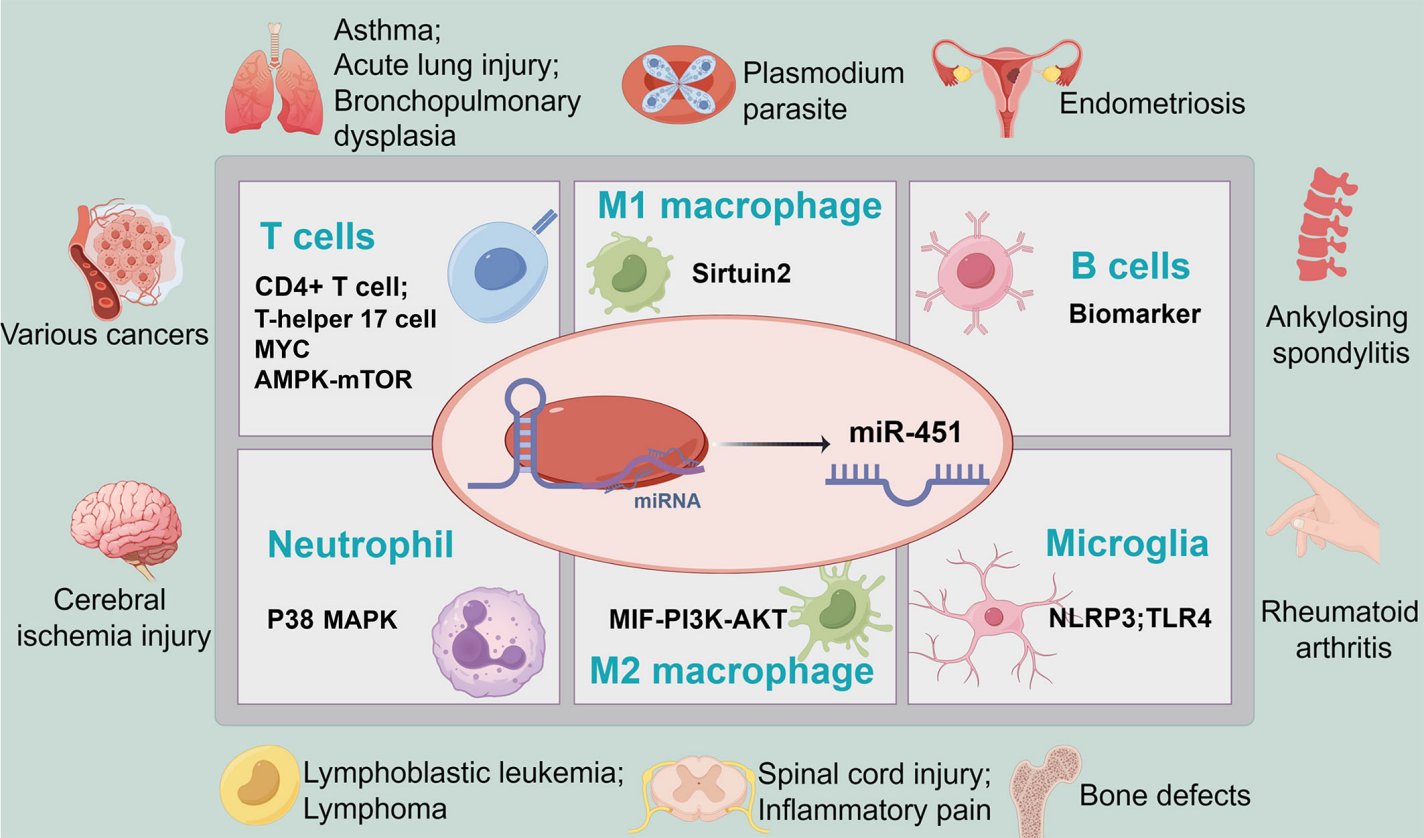
MiR-451 modulates the progression of diseases related to immune cells by interacting with various types of immune cells. See **Table 1** for abbreviations.

MicroRNAs (miRNAs) represent a class of endogenous small RNA molecules pivotal in modulating gene expression via post-transcriptional regulatory pathways. These molecules are extensively distributed throughout various bodily tissues and fluids, underscoring their integral role in cellular functions. The significance of miRNAs extends beyond basic cellular mechanisms to their utility as potential biomarkers for disease, thereby highlighting their importance in both foundational biomedical research and clinical translational applications (7, 8). In 2005, Altuvia et al. identified the gene encoding miR-451 within the human pituitary gland, pinpointed to the 17q11.2 region on the human chromosome. This landmark discovery has since propelled miR-451 to the forefront of scientific research, underscoring its critical role in a myriad of physiological and pathological contexts (9). Subsequent investigations have elucidated miR-451’s capacity to modulate an array of immune cells, thus playing a consequential role in disease progression. Specifically, miR-451’s regulatory influence spans across several immune cell types, including but not limited to microglia, macrophages, and neutrophils (10–12) (**Table 1**). This review aims to delve into the intricate interplay between miR-451 and immune cells, seeking to shed light on miR-451’s regulatory mechanisms within immune responses and explore its viability as a therapeutic target for relevant diseases (**Figure 1**).

**Table 1:** Impact of miR-451 on various immune cell-associated disorders and target interactions.

Disease model	Function	Targets	References
Spinal cord injury	Diminution of secondary neuronal inflammation post-spinal cord injury	NLRP3	(10)
Acute lung injury	Modulation of macrophage M2 polarization to mitigate acute lung injury	MIF-PI3K-AKT	(11)
Plasmodium parasite infection	Suppression of CD4+ T cell proliferation	MYC	(13)
Gastric cancer	Augmentation of T-helper 17 cell differentiation	AMPK/mTOR	(14)
T cell acute lymphoblastic leukemia	Inhibition of T-cell Acute lymphoblastic leukemia progression	MYC	(15)
Pediatric precursor B-cell acute lymphoblastic leukemia/Diffuse large B-cell lymphoma	Biomarker identification	N/A	(16–18)
Various cancers	Attenuation of tumor progression and amplification of therapeutic response	MIF	(19–25)
Ankylosing spondylitis	Suppression of inflammatory response in ankylosing spondylitis	MIF	(26)
Endometriosis	Enhancement of endometrial epithelial cell survival	MIF	(27)
Cerebral ischemia injury	Regulation of angiogenesis via human umbilical vein endothelial cells	MIF	(28)
Bronchopulmonary dysplasia	Facilitation of bronchopulmonary dysplasia development	MIF	(29)
Asthma	Contribution to allergic CCL17 induction in the lung and central role in pro-asthmatic macrophage activation	Sirtuin2	(30)
Bone defects	Promotion of bone healing	MIF	(31)
Rheumatoid arthritis	Reduction of neutrophil chemotaxis	p38 MAPK	(32)
Inflammatory pain	Inhibition of microglia-induced inflammatory response and alleviation of inflammatory pain	TLR4	(33)

AKT, protein kinase B; AMPK, AMP-activated protein kinase; MAPK, mitogen-activated protein kinase; MIF, macrophage migration inhibitory factor; mTOR, mechanistic target of rapamycin; MYC, myelocytomatosis oncogene; N/A, not applicable or not available; NLRP3, NOD-like receptor protein 3; PI3K, phosphoinositide 3-kinase; TLR4, toll-like receptor 4.

## 2 miR-451 family

The miR-451 gene family is comprised of two primary members: hsa-miR-451a and hsa-miR-451b, also known as MIR451a and MIR451b. These are located on chromosome 17 at positions chr17: 28861369-28861440 and 28861371-28861438, respectively. Both genes exhibit mature sequences of 22 nucleotides, displaying significant evolutionary conservation. Situated 100 bp downstream of the miR-144 gene, the miR-451 gene is positioned in intergenic regions adjacent to the Era G-protein-like 1 (ERAL1) gene. ERAL1 encodes a protein and is transcribed in the reverse orientation relative to the miR-144/451 gene cluster. The conservation of the miR-451 gene is apparent in a range of vertebrates, including humans, zebrafish, and mice. Within the miR-451 cluster, alongside miR-144 and miR-4732, unique sequences are identified, delineating distinct target sets for each miRNA. This specificity underscores their critical roles in gene expression regulation, reflecting their significance in biological processes and potential influence on disease pathogenesis and therapeutic strategies (34, 35).

Given the modulatory effects of miR-451 on diverse immune cell populations and its implications for disease progression, our present review will delve deeper into the specific regulatory mechanisms exerted by miR-451 on distinct immune cell subsets, informed by current research findings. Additionally, we will explore its potential as a diagnostic biomarker for immune-related diseases.

## 3 T cells

The immune system, a pivotal defense mechanism in organisms, encompasses an array of biological structures and intricate processes aimed at countering external infections, as well as aberrant and transformed cells. Within higher organisms, the immune system bifurcates into two fundamental branches: innate and adaptive immunity (36). The activation of adaptive immunity, in contrast to innate immunity, is prolonged and intricately tied to the precise activation of T and B lymphocytes, demonstrating exceptional specificity and immunological memory capabilities. The interaction between the T cell antigen receptor complex and peptide antigens presented by antigen-presenting cells instigates T cell activation through intercellular signaling, leading to the differentiation into the primary T cell subsets, CD8+ and CD4+ T cell (37). Studies have revealed that pivotal processes in T cell biology, including activation, differentiation, and the fulfillment of effector functions, are closely interconnected with alterations in cellular metabolic pathways (38).

A wealth of research has established a significant link between miRNAs and T cells, highlighting their ability to modulate T cell functionality, differentiation, and polarization through sequence-specific interactions that regulate multiple mRNA targets at the post-transcriptional level (39, 40). Moreover, microRNA-based therapies hold promise for refining T cell receptor signaling, augmenting T cell longevity, and enhancing effector functions, thereby setting the stage for advanced adoptive immunotherapy approaches (41).

The impact of malaria on public health is profound, with the activation and proliferation of T cells playing a crucial role in curbing the progression of the disease. Research has demonstrated that miR-451 inhibits the proliferation of CD4+ T cells following malaria infection in mice. This inhibitory effect is partly mediated through a mechanism dependent on MYC, which is a key target of miR-451 and plays an essential role in cell cycle regulation and cellular proliferation (13). Notably, miR-451’s expression markedly decreases in gastric cancer tissues yet increases within infiltrating T cells and exosomes. Crucially, miR-451 within exosomes not only predicts a poorer prognosis for patients undergoing gastric cancer surgery but also facilitates the differentiation of Th17 cells in the gastric cancer milieu (14). Furthermore, miR-451 functions as a suppressor, halting the progression of T-cell acute lymphoblastic leukemia (15). Remarkably, expression levels of miR-451 in patients with adult T-cell leukemia/lymphoma, EBV-induced lymphoma, rheumatoid arthritis, and anaplastic large cell lymphoma differ significantly from those in healthy individuals, positioning miR-451 as a promising therapeutic and diagnostic target for these conditions (42–46). Ongoing research aims to elucidate more fully the dynamics between miR-451 and T cells and explore its therapeutic and diagnostic potential in these diseases.

## 4 B cells

B cells play a crucial role in the adaptive immune system, primarily orchestrating humoral immune responses (37, 47). Their primary function involves the production of antibodies and the specific recognition and binding of foreign antigens, such as the surface proteins of bacteria and viruses, thereby acting as protectors of the organism (48–50). Beyond their central role in humoral immunity, B cells also perform various other functions necessary for mediating and regulating immune homeostasis. Importantly, B cells are indispensable for initiating T cell immune responses, where antigen-specific interactions between B and T cells may require antigen internalization and processing by the B cell receptor (BCR), followed by presentation to T cells in an MHC-restricted manner. The development or dysregulation of B cells is closely associated with the onset of various diseases, including autoimmune diseases, immunodeficiency disorders, and tumors, such as hematological malignancies (51).

Investigations have elucidated that miRNAs are crucial in orchestrating the development of bone marrow B cells, as well as in establishing populations within the peripheral immune system. The BCR serves as a fundamental regulator for the development, differentiation, and functionality of B cells. Following BCR signaling, three essential pathways are activated upon BCR engagement: the NF-κB pathway, the RAS-MAPK pathway, and the PI3K-AKT pathway. A multitude of miRNAs, including, but not limited to, miR-185, miR-30, miR-29, miR-146a, and miR-155, are instrumental in modulating the development, differentiation, and function of B cells through these principal signaling cascades (52). Additionally, in the realm of oncology, certain miRNAs, such as miR-17-92 cluster, miR-146a, and miR-155, along with their regulatory networks, have been pinpointed as potential molecular intermediaries linking autoimmunity with B cell malignancies. Moreover, autoimmune conditions are believed to foster a conducive microenvironment for cancer progression. Considering the plausible association between autoimmunity and cancer, particularly under conditions of miRNA regulatory imbalance, pursuing comprehensive research in this area is deemed critically significant (53).

The relationship between miR-451 and B cells is primarily centered on its role as a biomarker for associated diseases, particularly in the context of hematologic disorders linked to B cells. Recent studies have demonstrated that in pediatric precursor B-cell acute lymphoblastic leukemia, there is a notable reduction in the expression levels of miR-451, underscoring its potential as a disease biomarker (54). This observation has received further validation through meta-analytic review (16). Additionally, research has highlighted that miR-451 not only serves a diagnostic purpose in pediatric precursor B-cell acute lymphoblastic leukemia but is also instrumental in predicting disease recurrence and aiding in the refinement of risk stratification at the time of diagnosis. This facilitates the initiation of early therapeutic interventions, ultimately improving the survival prospects for patients at high risk (17). Notably, in patients with diffuse large B-cell lymphoma, elevated miR-451 levels in the plasma of patients in complete remission have been shown to effectively distinguish between individuals with residual tumors and those in complete remission during remission evaluation (18). These findings lend support to the potential of miR-451 as a biomarker for B-cell-related diseases. Nevertheless, further comprehensive research is needed to explore additional interactions between miR-451 and B cells beyond its biomarker function, particularly its potential role in mediating the interactions between B cells and T cells.

## 5 Macrophage

Macrophages, a cell population extensively dispersed throughout the body, derive their diversity from various sources including embryonic and adult bone marrow, the specific tissue environments they occupy, microbial invasions, tissue injuries, metabolic imbalances, as well as the activation or deactivation of a myriad of signaling pathways, and their activation in response to adaptive T cell activities (55). These cells are capable of classical adaptive responses, such as exhibiting tolerance, engaging in activation processes, and entering states of heightened activity, broadly classified into the pro-inflammatory M1 phenotype and the anti-inflammatory M2 phenotype. Nonetheless, this categorization may be an oversimplification and warrants further exploration. Notably, macrophages with different phenotypes exhibit unique functions and transcriptional profiles, each equipped with unique abilities to eliminate pathogens and repair inflammation-induced damages, respectively. Macrophage plasticity is an integral aspect of chronic inflammation and contributes to the onset of a variety of human pathologies, especially cancer. The dynamic interplay among epigenetic mechanisms, transcription factors, and miRNAs networks underpins the macrophages’ adaptive capabilities to diverse environmental stimuli (55, 56). Consequently, modulating macrophage activation emerges as a pivotal strategy in the prophylaxis and therapeutic management of infectious diseases, inflammatory conditions, and tumors (57).

Extensive investigations have underscored the crucial role of miRNAs in regulating macrophage activities, with a significant emphasis on their impact on macrophage activation (58, 59). Notably, tumor-derived exosomes, such as miR-934, are known to promote liver metastasis in colorectal cancer by altering the interactions between colorectal cancer cells and tumor-associated macrophages. This finding elucidates the crucial mediating function of tumor-derived exosomes within the metastatic niche, profoundly influencing the dynamic interplay between tumor cells and tumor-associated macrophages, which, in turn, plays a significant role in the progression of liver metastasis in colorectal cancer (60). Additionally, miRNAs exert a considerable influence on the migration and phagocytic functions of macrophages. As an illustration, the deletion of miR-301a diminishes macrophage migration and phagocytosis through the YY1/CXCR4 signaling pathway (61). Despite the abundance of research, the complex interactions between miRNAs and macrophages demand further comprehensive exploration. Particularly, examining the distinct regulatory functions of macrophage-derived miRNAs versus those originating from other sources in influencing macrophage behavior represents a fascinating and important direction for future research.

The relationship between miR-451 and macrophages is principally centered on the macrophage migration inhibitory factor (MIF). A multitude of research endeavors has established a close correlation with an extensive spectrum of diseases, including neuroblastoma, ankylosing spondylitis, colorectal cancer, prostate cancer, osteosarcoma, gastrointestinal cancers, nasopharyngeal carcinoma, renal cell carcinoma, endometriosis, and cerebral ischemic injury (19–28). Moreover, miR451 contributes to the pathogenesis of conditions such as acute lung injury, bronchopulmonary dysplasia, stenosis in tissue-engineered vascular grafts, and asthma, by modulating macrophage migration, invasion, activation, among other mechanisms (11, 29, 30, 62). From a biomarker perspective, miR451’s significance is underscored by findings such as its low expression levels serving as a prognostic indicator for poor outcomes in non-small cell lung cancer patients (63). The dynamic interplay between reactive oxygen species and miR-451 in controlling the oxidative stress response in macrophages further highlights the intricate relationship (64). These discoveries collectively underscore the sophisticated and detailed interplay between miR451 and macrophages. Notably, macrophages are capable of expressing miR451, which in turn regulates their functional and activation states through its target, MIF, thereby establishing a feedback mechanism. The precise nature of this mechanism, whether facilitatory or inhibitory, necessitates further exploration. Specifically, the role of miR451-mediated interactions between MIF and tumor-associated macrophages, including their potential to either promote or inhibit tumor growth, represents a significant area for forthcoming studies. Delving deeper into these phenomena promises to shed light on new theoretical concepts and practical approaches for treating associated diseases.

## 6 Other immune cells

Beyond the key immune cells previously mentioned, there is emerging evidence of a link between miR-451 and various immune cells. Specifically, in cases of nasal natural killer/T-cell lymphoma, notable fluctuations in miR-451 expression levels have been observed. The connection between these fluctuations and the disease’s pathogenesis, however, remains to be clearly defined. Consequently, there is a pressing need for in-depth research to elucidate the relationship between these variables (43, 65). Furthermore, miR-451 has been shown to modulate the cytokine response of dendritic cells during influenza infection, potentially affecting the course of the disease (66). This insight sheds light on the critical role of miR-451 within the immune system, suggesting avenues for future investigation.

Neutrophils, complex cells with myriad crucial functions, play an integral role as effector cells in the innate immune response. They are involved in regulating diverse processes, from acute injury repair to cancer, autoimmunity, and chronic inflammation (67). Research has demonstrated that neutrophils from patients with rheumatoid arthritis have diminished miR-451 expression levels compared to healthy individuals, and that elevated expression of miR-451 markedly reduces neutrophil chemotaxis (32). Notably, another study found increased miR-451 expression in peripheral blood mononuclear cells of individuals predisposed to rheumatoid arthritis, proposing its utility as a biomarker for the condition (68). In the context of imatinib-resistant chronic myeloid leukemia, there is evidence of decreased miR-451 expression coupled with heightened MYC expression, suggesting a regulatory feedback loop that offers a potential therapeutic target (69). These insights reveal that miR-451 expression varies across different disease stages and among various immune cells, underscoring its versatility and potential as a biomarker. Furthermore, neutrophils are capable of producing miR-451. Investigations have shown that vesicles secreted by activated neutrophils, which include miR-142-3p and miR-451, are capable of targeting endothelial cells. This interaction initiates inflammatory cascades and leads to direct vascular damage (12), indicating that neutrophils can function both as sources and targets of miR-451. However, the precise mechanisms involved warrant further exploration.

The significance of miR-451 extends beyond its regulatory impact on circulating immune cells, it also influences tissue-resident macrophages. MiR-451 notably dampens the inflammatory response triggered by activated microglia through the suppression of Nucleotide-binding oligomerization domain-like receptor protein 3 expression (10). Moreover, by modulating Toll-like receptor 4, miR-451 further curtails the inflammatory response incited by activated microglia, thus mitigating inflammatory pain (33). In the context of glioblastoma, extracellular vesicles secreted by these tumors carry miR-21 and miR-451, which are capable of being assimilated by microglia and monocytes/macrophages, thereby fostering their proliferation. This finding has been validated through visualization studies (70, 71). Nonetheless, the precise regulatory roles of miR-451 on additional immune cell types necessitate further investigation.

## 7 Conclusions and perspectives

The regulatory role of miR-451 in immunoregulation is distinguished by its diverse effects on various immune cell types, dependent on the disease context and specific cell type involved. Immune cells are capable of secreting extracellular vesicles that contain miR-451, which in turn modulates the functions of both similar immune cells and other target cells, such as endothelial cells (12). The mechanisms underlying miR-451 production and its feedback loop with immune cells, which could be either positive or negative, and its implications for disease progression, warrant further detailed investigation. Moreover, the mechanisms by which miR-451 exerts its regulatory effects on different immune cells—whether directly targeting each type of immune cell, indirectly affecting other immune cells through modulation of a specific cell type, or through a combined approach—require deeper examination. In addition to its immunoregulatory functions, the potential of miR-451 as a biomarker for immune-related diseases, facilitated by its presence in extracellular vesicles, is significant. This opens up promising opportunities for identifying new therapeutic targets and developing intervention strategies for these diseases. However, extensive and rigorous research is essential to confirm this potential.

Furthermore, the diversity and complexity of immune cells underscore the immune system’s capacity to meet various challenges. Through comprehensive investigation into the functionalities and interplays among these cells, researchers can develop novel therapeutic approaches, including vaccines, immunomodulators, and cancer immunotherapies. Such advancements contribute significantly to improving human health and enhancing the efficacy of disease prevention and treatment. Ongoing studies in immune cell research consistently emphasize the crucial role of epigenetic mechanisms, such as miRNAs, in regulating immune cell behavior. As scientific and technological progress continues, it is expected that additional cellular types and their functions will be identified, offering fresh insights and approaches for both immunological research and clinical practice.

## Author contributions

F-XW: Writing – original draft, Writing – review & editing. Z-AS: Writing – original draft, Writing – review & editing. GM: Conceptualization, Methodology, Writing – review & editing.
